# A171 INTESTINAL INTERFERON-LAMBDA RECEPTOR 1 EXPRESSION AND RESPONSES ARE SIGNIFICANTLY DECREASED IN PEDIATRIC INFLAMMATORY BOWEL DISEASE PATIENTS

**DOI:** 10.1093/jcag/gwac036.171

**Published:** 2023-03-07

**Authors:** O Ogungbola, R Mahmood, K Bittorf, N Nguyen, W El-Matary, E Wine, L Tyrrell, H Armstrong, D Santer

**Affiliations:** 1 University of Manitoba, Winnipeg; 2 University of Alberta, Edmonton, Canada

## Abstract

**Background:**

While interferon-lambdas (IFN-λs) were initially discovered for their role in antiviral immunity at mucosal barriers such as the lung and gut, there are many unanswered questions for how IFN-λs uniquely dampen inflammatory immune responses. In mouse colitis models, IFN-λs were shown to play a significant role in promoting epithelial barrier integrity and mucosal healing. Microbes present in the gut naturally induce IFN-λs, but how chronic inflammation, such as in patients with inflammatory bowel diseases (IBD), affects IFN-λ biology has not been well studied, especially in humans.

**Purpose:**

Here, we tested the hypothesis that children with active, more severe IBD would present with lower intestinal IFN-λR levels and IFN-λ responses which could contribute to IBD pathology through exacerbated inflammation, decreased mucosal healing and impaired barrier function.

**Method:**

We screened 14 novel antibodies to find the optimal clone that accurately stains the unique IFN-λ receptor subunit (IFN-λR1) protein in human intestinal tissue and IFN-λR1 levels were quantified by immunohistochemistry and flow cytometry in biopsy samples from children without IBD, Crohn’s disease, or ulcerative colitis (n=35 total). Fresh patient biopsies (ascending colon or terminal ileum) were also treated in media +/- IFN-λ for 24hrs using our novel *ex vivo* biopsy assay and changes in gene expression were quantified by RT-qPCR. Intestinal washes were also collected and microbes profiled by shotgun metagenomics.

**Result(s):**

We identified 2 new antibodies that accurately stained human cell lines and immune cells known to express IFN-λR1 protein (gut epithelial cells and B cells). We found that IFN-λ receptor (IFN-λR) levels are significantly reduced in gut epithelial and immune cells within pediatric IBD intestinal tissue, even at non-inflamed sites (p<0.01, 30-50% reduction) and this was even more pronounced when comparing moderate/severe disease compared to children with no disease activity. This led to lower IFN-λ responsiveness in IBD compared to non-IBD biopsies when investigating IFN-stimulated gene expression (p<0.05, up to 7-fold reduction). Paired patient gut microbe analyses identified specific species that correlated with changes in IFN-λ receptor expression.

**Conclusion(s):**

Together, our findings demonstrate pediatric IBD patients may be less able to induce critical IFN-λ-mediated antimicrobial responses and protective anti-inflammatory pathways. This work supports the goal to restore and promote IFN-λ responses as a novel therapeutic strategy for pediatric IBD.

**Please acknowledge all funding agencies by checking the applicable boxes below:**

Other

**Please indicate your source of funding;:**

Children's Hospital Research Institute of Manitoba

**Disclosure of Interest:**

None Declared

